# Genotypic Characterization of *Infectious Spleen and Kidney Necrosis Virus* (ISKNV) in Southeast Asian Aquaculture

**DOI:** 10.1155/2023/6643006

**Published:** 2023-03-21

**Authors:** Cahya K. Fusianto, Joy A. Becker, Kuttichantran Subramaniam, Richard J. Whittington, Samantha A. Koda, Thomas B. Waltzek, Murwantoko, Paul M. Hick

**Affiliations:** ^1^School of Life and Environmental Sciences, The University of Sydney, 380 Werombi Road, Camden, NSW 2570, Australia; ^2^Sydney School of Veterinary Science, The University of Sydney, 425 Werombi Road, Camden, NSW 2570, Australia; ^3^Department of Infectious Diseases and Immunology, College of Veterinary Medicine, University of Florida, Gainesville, FL 32611, USA; ^4^Department of Fisheries, Faculty of Agriculture, Universitas Gadjah Mada, Jl. Flora 1, Bulaksumur, Sleman 55281, Yogyakarta, Indonesia

## Abstract

*Infectious spleen and kidney necrosis virus* (ISKNV) is a species within the genus *Megalocytivirus* (family Iridoviridae), which causes high mortality disease in many freshwater and marine fish species. ISKNV was first reported in Asia and is an emerging threat to aquaculture with increasing global distribution, in part due to its presence in ornamental fish with clinical and subclinical infections. The species ISKNV includes three genotypes: red seabream iridovirus (RSIV), turbot reddish body iridovirus (TRBIV), and ISKNV. There is an increasing overlap in the recognized range of susceptible fish hosts and the geographic distribution of these distinct genotypes. To better understand the disease caused by ISKNV, a nucleic acid hybridization capture enrichment was used prior to sequencing to characterize whole genomes from archived clinical specimens of aquaculture and ornamental fish from Southeast Asia (*n* = 16). The method was suitable for tissue samples containing 2.50 × 10^4^–4.58 × 10^9^ ISKNV genome copies mg^−1^. Genome sequences determined using the hybridization capture method were identical to those obtained directly from tissues when there was sufficient viral DNA to sequence without enrichment (*n* = 2). ISKNV genomes from diverse locations, environments, and hosts had very high similarity and matched established genotype classifications (14 ISKNV genotype Clade 1 genomes with >98.81% nucleotide similarity). Conversely, two different genotypes were obtained at the same time and location (RSIV and ISKNV from grouper, Indonesia with 92.44% nucleotide similarity). Gene-by-gene analysis with representative ISKNV genomes identified 59 core genes within the species (>95% amino acid identity). The 14 Clade 1 ISKNV genomes in this study had 100% aa identity for 92–105 of 122 predicted genes. Despite high overall sequence similarity, phylogenetic analyses using single nucleotide polymorphisms differentiated isolates from different host species, country of origin, and time of collection. Whole genome studies of ISKNV and other megalocytiviruses enable genomic epidemiology and will provide information to enhance disease control in aquaculture.

## 1. Introduction

*Infectious spleen and kidney necrosis virus* (ISKNV) is a species within the genus *Megalocytivirus* (MCV) (subfamily *Alphairidovirinae*; family *Iridoviridae*) that encompasses several emerging pathogens of finfish [1]. The ISKNV genotype was first reported as a cause of disease in freshwater mandarin fish (*Siniperca chuatsi*) cultured in China in the 1990s [2]. Red sea bream iridovirus (RSIV) is now recognized as a genotype of the same virus species and caused mass mortality in multiple species of marine fish, including red sea bream (*Pagrus major*) in Japan, since 1990 [3, 4]. Fish infected with ISKNV develop histopathological lesions characterized by megalocytes and enlarged cells containing basophilic cytoplasmic inclusions in connective and perivascular tissues across various organs (reviewed in [5]). The disease manifests with nonspecific clinical signs, including anorexia, lethargy, gill pallor, skin discoloration, splenomegaly, and internal or external haemorrhages [6–11]. Fish of all ages can be affected with mortality from 20%–100% in aquaculture settings [9, 12, 13]. In addition to clinical disease, ISKNV has been reported to cause subclinical infection in a variety of hosts and environments [14, 15]. The broad host range of ISKNV includes over 50 species of fish spanning 13 families of the order Perciformes including marine and freshwater fish [16, 17].

Megalocytiviruses have a hexagonal nucleocapsid measuring 115 to 200 nm diameter that encloses a linear double-stranded DNA genome which is 110–112 kb [1]. Phylogenetic characterization of ISKNV based on the major capsid protein (MCP) gene and the ATPase gene has identified a consistent differentiation between three genotypes named ISKNV, RSIV, and turbot reddish body iridovirus (TRBIV) [10]. These genotypes and two clades within each have been supported by analysis of increasing numbers of complete ISKNV genomes, providing a sound nomenclature for the classification of isolates within the species [18].

The continued emergence of ISKNV is highlighted by a breakdown in the distinct host-geographical niche that was once attributed to the three genotypes. The RSIV genotype associated with marine fishes in Japan [7] was also detected in Korea [19] and China [20]. Subsequently, the range has expanded with RSIV-related diseases in marine fish in the Dominican Republic [18], barramundi (*Lates calcarifer*) in estuary-based cages in India [21], and the freshwater mandarin fish in China in locations previously impacted by the ISKNV genotype [22]. The ISKNV genotype identified initially in freshwater aquaculture [6] is globally widespread, impacting freshwater and marine fish in Asian countries including China [23], India [24], Indonesia [15, 25–27], Malaysia [28, 31], Vietnam [11], and Thailand [30]. Reports of the increasing global distribution of the ISKNV-genotype include North America [31], South America [32], Africa [33], Europe [34], and Australia [14, 35]. In recent years, there has also been an expansion of the host range for ISKNV, most notably affecting several important aquaculture sectors. For example, tilapia (*Oreochromis niloticus*) is a freshwater species farmed in 127 countries; it is critical to domestic food security in many developing countries and represents just over 5% of global aquaculture production [36]. Epidemics due to ISKNV have been recorded in farmed tilapia in the USA [31], Africa [33], and Thailand [37]. Although TRBIV was initially considered to be restricted to flatfish in China and South Korea [8], it has been reported in retrospective studies of ornamental fish as early as 1986 [10, 38]. More recently, TRBIV was the cause of disease in barramundi resulting in severe mortality (up to 90%) at 35 farms in Taiwan [39].

The World Organization for Animal Health (WOAH) recognized the importance of controlling disease caused by ISKNV by listing red sea bream iridoviral disease as notifiable, under a definition including infection with RSIV and ISKNV-like viruses [17]. There are several barriers restricting the measures to minimize the spread and disease impacts of ISKNV. First, the nomenclature is confusing, with many virus names reported using the host fish and iridovirus descriptor without reference to the ISKNV species designation [40]. This excessive array of virus names within ISKNV overlaps with viruses from different genera such as *Singapore grouper iridovirus* (genus *Ranavirus*). Meanwhile, the generic term “megalocytiviruses” to refer to all genotypes of ISKNV, e.g., Crane and Moody [16] is inappropriate with the emergence of another pathogenic megalocytivirus species, *Scale drop disease virus* (SDDV) [41]. Second, there has been limited validation of high throughput diagnostic tests suitable for the identification and differentiation of each genotype of ISKNV [17]. Third, ISKNV is frequently refractory to isolation in cell culture which limits the options for the characterization of isolates. Improved disease control and effective policy to limit the spread of ISKNV require further evaluation of the genomic diversity within the ISKNV species. Genomic epidemiology can evaluate the pathways for the spread of ISKNV that contribute to the numerous reports describing the first detections of ISKNV in new hosts and locations (e.g., Pereira Figueiredo et al. [42] and Sukenda et al. [27]). Evaluation of the complete genome of ISKNV could also identify factors that determine host and geographical range and influence viral phenotype, thereby influencing the spectrum of clinical outcomes from subclinical infection to severe disease [45].

The objective of this study was to evaluate a hybridization capture nucleic acid enrichment method to determine the complete genome sequence of ISKNV directly from clinical specimens and to evaluate the genomic diversity of ISKNV in archived specimens representing a broad range of hosts, locations, and clinical status for fish in Southeast (SE) Asia. Improved understanding of the genomic diversity of ISKNV and other megalocytiviruses is important to understand the disease and informing disease control measures including biosecurity and vaccination.

## 2. Methods

### 2.1. Samples of ISKNV-Infected Fish

Specimens were recruited into the study to represent the diversity of ISKNV in the laboratory archive collected from SE Asian locations between 2011 and 2018 (Table 1). Samples were selected to maximize diversity with respect to the country of origin, host species, and year of collection and to compare samples from related disease outbreaks in grouper aquaculture. Most of the samples in the current study were archived as nucleic acids that were purified from fish with ISKNV infection identified using the qPCR described by Rimmer et al. [49] and stored at −80°C (*n* = 12). These had been prepared by homogenizing visceral tissues by bead beating and were extracted using the MagMax-96 Viral Isolation Kit (ThermoFisher) according to the directions of the manufacturer (Samples 1–11 and 16). An additional 4 samples were prepared from visceral tissues preserved in 80% ethanol (Samples 12–15) using a phenol-chloroform precipitation method for nucleic acid purification described later in the article [50].

### 2.2. Purification and Quantification of Nucleic Acids

Nucleic acids were treated to remove RNA using 4 *µ*l of DNase-free RNase A (2 *µ*g/ml, Qiagen) with incubation at 37°C for 1 hour and then purified by ethanol precipitation. Briefly, for each volume of the nucleic acid preparation, 1/20 volume of 3 M sodium acetate and 3 volumes of absolute ethanol were added, followed by overnight incubation at −20°C. Samples were centrifuged at 12,000 × *g* for 20 min, the supernatant was discarded, and 500 *µ*L of 70% ethanol (at −20°C) was added, followed by centrifugation at 12,000 × *g* for 10 min. The ethanol was removed by pipetting followed by air-drying, and the DNA pellet was resuspended in 100 *µ*L of Tris-EDTA buffer (pH 8). For the 4 samples obtained as ethanol preserved tissues, a 0.5 g pool of equal parts liver, spleen, and kidney was air-dried and washed with phosphate buffered saline (PBS). The pooled tissues were homogenized by grinding using plastic pestles in 360 *µ*l RLT lysis buffer (Qiagen). Proteinase K was added (50 *µ*l of 20 *µ*g/ml; Sigma-Aldrich) with overnight incubation at 56°C for enzymatic digestion before performing RNase treatment as previously described. Phenol:chloroform:isoamyl-alcohol (25 : 24 : 1) was added (400 *µ*l), and the samples were incubated at an ambient temperature for 15 min in a fume hood. The aqueous layer was collected after centrifugation at 2,000 × *g* for 10 min and DNA was obtained by ethanol precipitation as previously described.

The purity and quantity of the DNA in all samples were measured using a Qubit® dsDNA BR Assay Kit (Invitrogen) and NanoDrop spectrophotometer 1000 (Thermo Fisher Scientific). The quantity of ISKNV DNA was determined using a quantitative real-time PCR (qPCR) according to the method described by Rimmer et al. [49]. Briefly, individual samples were tested in duplicate 25 *μ*L reactions containing 12.5 *µ*l of Quantitech SYBR Green Master Mix (Qiagen), 250 nM each of the forward (C1073) and reverse (C1074) primers, 2.5 *µ*l of template DNA, and molecular biology grade water. Control samples tested at the same time were: a tissue homogenate from an ISKNV-infected fish, negative extraction control, and no template control (nuclease-free water). A standard curve was prepared by amplification of the standard pDGIV-MCP1, which contained the MCV major capsid protein gene sequence in preparation for linearized plasmid DNA [49]. For each qPCR plate, duplicate reactions were prepared from a 7-step, 10-fold dilution series containing 10^1^ to 10^7^ copies of the standard in molecular grade water. Assays were run using an Mx3000P Multiplex Quantitative PCR System (Stratagene) under the following conditions: 1 cycle of initial denaturation at 95°C for 15 minutes, 40 cycles of denaturation at 95°C for 30 s, annealing at 62°C for 30 s, and extension at 72°C for 30 s, with fluorescence detection at the end of the annealing step. A dissociation curve was determined after the amplification cycles by heating the reaction products to 95°C for 1 min, annealing at 55°C for 30 s, and then heating to 95°C at a rate of 1°C every 30 s. A fluorescence threshold was determined by the Mx3000p software (Stratagene) based on the amplification of the plasmid standard and applied to the experimental samples. A threshold cycle (Ct) value was assigned based on an exponential increase in the SYBR fluorescence above the threshold when the melting temperature of the product was ±0.5°C of the positive control. A valid PCR run was defined by amplification of both replicates of the positive control with a cycle threshold (Ct) within the range of the standard curve (*r*^2^ > 0.95 and efficiency between 90 and 110%) and no amplification of negative controls. The quantification of viral DNA in positive samples was determined by interpolation from the plasmid DNA standard curve.

### 2.3. Design of Hybridization Bait Capture Sequences

A custom hybridization capture panel (SureSelect, Agilent) was designed for targeted enrichment of the whole genome of multiple genotypes of ISKNV. The design was based on 12 published ISKNV genomes: ISKNV (NCBI Acc number: AF371960 and KT781098); RSIV (AB104413, AP017456, AY779031, KT804738, KC244182, AY894343, and AY532606); and TRBIV (GQ273492, MG570132, and MG570131) and to meet the size limit for the SureselectXT custom 1 Kb–499 Kb library (Agilent). These genomes were aligned using MUSCLE in MEGA X [51]. Regions of the genome alignment in which the nucleotide similarity was greater than 90% were defined as conserved for the purpose of probe design. Regions of alignment with <90% nucleotide sequence similarity in a region of 180 bp or more were considered a variable region. Hybridization capture probes (120 bases) were designed to tile across the conserved regions with 2*x* coverage. Additional probes were designed for each genotype within the variable regions to achieve 2*x* coverage of each unique genotype (Supplementary Figure 1). The hybridization capture probes were designed for a melting temperature between 60–65°C and evaluated *in silico* by mapping to the positive strand of each ISKNV reference genome using CLC Genomic Workbench 12 (Qiagen) with similarity and length fraction 0.9 (Supplementary Table 1). The bait library oligonucleotide probes were manufactured by Agilent Technologies.

### 2.4. Sequencing Library Preparation, Hybridization, and Sequencing

Sequencing library preparation and hybridization were performed at the Westmead Institute of Medical Research, Genomics Facility. Libraries were generated using the SureselectXT HS Target Enrichment System for Illumina-Paired End Multiplexed Sequencing (Agilent Technologies) according to the instructions of the manufacturer. In brief, up to 200 ng of DNA for each sample was ultrasonically fragmented to 150–200 bp in 130 *µ*l microtubes (Covaris) using the Covaris E220 Evolution system. The following shearing parameters were used: peak incident power = 140 W; duty factor = 10%; cycles per burst = 200; treatment time = 100 s. End repair and A-tailing were performed by adding ligation buffer (207 *µ*l), T4 DNA ligase (18 *µ*l), end repair-A Tailing buffer (144 *µ*l), and enzyme mix (38 *µ*l) with incubation at 20°C for 15 min, and then 72°C for 15 min before being held at 4°C. Molecular barcode adapters were added by ligation at 20°C for 30 min. All incubation and PCR steps were performed using a Miniamp thermocycler (ThermoFisher). Samples were purified using Ampure XP beads (Beckman Coulter) with a Dynamag-96 side magnet (Thermo Fisher) according to directions to capture oligonucleotide >100 bp. The purified, adapter-ligated libraries were amplified by a precapture PCR protocol with the master mix composition: 180 *µ*l 5 × Herculase II reaction buffer, 9 *µ*l 100 mM dNTP Mix, 36 *µ*l forward primer and 18 *µ*l Herculase II fusion DNA polymerase. The thermocycling program depended on the quantity of input DNA: 98°C for 2 min, 11 cycles of 98°C for 30 s, 60°C for 30 s, and 72°C for 1 min for samples with 100–200 ng input DNA. For samples with a low input of DNA (50 ng), 12 cycles were used with final elongation at 72°C for 5 min. Amplified libraries were purified using Ampure XP beads, followed by fragment size and concentration assessment on a Tapestation 4200 (Agilent) using the D1000 tape.

Prepared libraries were hybridized to the target-specific capture library using the Sureselect XT HS Target Enrichment System for Illumina-Paired End Multiplexed Sequencing (Agilent Technologies) and the protocol for a target size <3000 base pairs. Briefly, 5 *µ*l of Sureselect XT HS Blocker Mix was added to prepared libraries and processed with a thermocycler using the following program: 95°C for 5 min, 65°C for 10 min, 65°C for 1 min, 60 cycles of 65°C 1 min, and 37°C 3 s, held at 65°C. The thermal cycling was paused at 65°C before entering the 60 cycles segment for the addition of 18 *µ*l of 25% RNase Block solution prepared as 4.5 *µ*l SureSelect RNase Block with 13.5 *µ*l nuclease-free water (NFW), 18 *µ*l Capture Library for <3 Mb kit, 54 *µ*l SureSelect hybridization buffer, and 27 *µ*l NFW and the thermal cycling process was continued. After the thermal cycler reached the 65°C hold step, the samples were transferred at room temperature to wells containing 200 *µ*l of washed Dynabeads MyOne Streptavidin T1 magnetic beads (ThermoFisher). These were prepared earlier according to the directions of the manufacturer to capture the hybridized DNA for purification using a magnetic separator.

Libraries enriched for the ISKNV target were washed using SureSelect Wash Buffers 1 and 2 according to directions before the post-capture PCR was used for amplification. The protocol for libraries <0.2 Mb required a reaction mix consisting of 250 *µ*l NFW, 180 *µ*l 5 × Herculase II reaction buffer, 18 *µ*l Herculase II Fusion DNA Polymerase, 9 *µ*l 100 mM dNTP Mix, and 18 *µ*l SureSelect Post-Capture Primer Mix. The thermocycling conditions were as follows: 98°C for 2 min; 14 cycles of 98°C for 30 s, 60°C for 30 s, and 72°C for 1 min; 72°C for 5 min. Amplified libraries were purified using Ampure XP beads, and final analysis of library size and concentration was performed on the Tapestation 4200. With the genome size 110 to 112 kb and expected depth of coverage of 1000 reads/nucleotide, sequencing of captured libraries was performed on an Illumina Miseq using the 300-cycle V2 kit for 150 bp paired end reads according to the manufacturer's protocol (Illumina, US).

### 2.5. Genome Assembly and Annotation

Removal of the Illumina adaptor sequences from raw sequence files and filtering of the low-quality reads (minimum Phred score of 20) was performed using Trimmomatic [52]. The trimmed FASTQ files were paired using the FASTQ joiner [53]. *De novo* assembly of the paired and unpaired reads was performed with SPAdes Genome Assembler Version 3.14.1 [54] with correction on mismatches and short indels and K-mer values set to 21, 33, and 55. Genome orientation and completeness were evaluated by comparison to the published ISKNV reference genomes based on genome length, read coverage, GC content (%), and pairwise comparison after progressive alignment using CLC Genomic Workbench 12.

The Genome Annotation Transfer Utility (GATU) was used to annotate assembled genomes [55]. The annotated genes and other predicted ORFs identified by GATU were included using the following criteria: (1) larger than 120 nucleotides, (2) not overlapping with another ORF by more than 25%, and (3) in the case of overlapping ORFs, only the larger ORF was annotated [38]. Four reference genomes were used for annotation references: Angelfish Iridovirus (AFIV-16) (MK689685.1) for the ISKNV genotype samples and Pompano iridovirus strain PIV2014a (MK098186.1) and RSIV strain KagYT-96 (MK689686.1) for the RSIV genotype sample.

### 2.6. Genetic and Phylogenetic Analyses

The complete genomes were aligned using progressive MAUVE [56] including AFIV-16 for comparison. Dot plots were generated using JDotter [57] with AFIV-16 on the horizontal axis for the ISKNV genotype and PIV2014a for the RSIV genotype. Gene-to-gene similarity comparison was conducted using GATU with AFIV-16 as the reference sequence for the ISKNV genotypes and RSIV strain KagYT-96 for Sample 16 (RSIV). Repeated sequences were identified through the tandem repeats finder (https://tandem.bu.edu/trf/trf.submit.options.html) [58].

Core genes of ISKNV species were identified by comparing multiple whole genomes for the ISKNV genotype (AFIV-16 MK689685, ISKNV AF371960, ISKNV_KU1 MT128666, ISKNV_KU2 MT128667, ISKNV_ RSIV-Ku KT781098, ISKNV_EFIV-2019 MW273354, and ISKNV_EFIV-2018 MW273353), RSIV genotype (RSIV_RIE12-1 AP017456, PIV2014a MK098186, RSIV strain KagYT-96 MK689686, GSIV-K1 KT804738, RBIV-C1 KC244182, RBIV-KOR-TY1 AY532606, and OSGIV AY894343), and TRBIV genotype (TRBIV GQ273492, TSGIV MG570132, and SACIV MG570131). Gene-to-gene comparison was performed using GATU, which used NEEDLE [59] to obtain similarity at the amino acid level. Genes with a similarity ≥95% for the three genotypes were defined as ISKNV core genes.

Each core gene was subject to BLASTP (https://blast.ncbi.nlm.nih.gov) analysis against the GenBank nonredundant protein database to identify orthologous sequences. The amino acid sequence for each core gene was aligned using ClustalW and concatenated into a single alignment using CLC Genomic Workbench 12. A maximum likelihood phylogenetic tree was generated in IQ-TREE [60] with the best-fit amino acid substitution model determined using ModelFinder [61] and 1000 bootstraps through ultrafast bootstrap approximation approach [62].

A single nucleotide polymorphism (SNP) alignment was built using a CSI Phylogeny 1.4 web tool with a default setting (https://cge.cbs.dtu.dk/services/CSIPhylogeny/ [63]. IQ-TREE was used for phylogenetic analysis of this SNP alignment as previously described. Geographic visualization was conducted using Mapbox Studio (https://studio.mapbox.com).

### 2.7. Comparison of Hybridization Capture Enrichment with Direct Sequencing of Clinical Specimens

The hybridization capture enrichment method was compared with a direct Illumina MiSeq approach to sequencing nucleic acids purified from the clinical specimens according to the method described by Fusianto et al. [47]. Two samples with the highest load of ISKNV were suitable for determining the whole genome by direct sequencing and were used for the comparison (Samples 12 and 15). The number of mapped ISKNV-specific reads, depth of coverage, genome coverage, and identification of SNPs was compared for both methods using CLC Genomic Workbench 12.

## 3. Results

### 3.1. Hybridization Bait Capture Design

The library design contained probes that targeted conserved regions (589) and the less conserved regions of each genotype, i.e., ISKNV (942), RSIV (946), and TRBIV (244). The number of probes for the less conserved regions of TRBIV was constrained by the library size that was used. *In silico* mapping of the probe library to 12 reference genomes indicated that the proportion of the genomes targeted by probes for conserved and variable regions was 42% and 58%, respectively. The average predicted coverage of reference genomes by the probes was 1.98 to 2.29 times (Supplementary Table 1). There were 70 regions of zero probe coverage for intervals between 1 and 299 bp for TRBIV (Supplementary Figure 1).

### 3.2. Evaluation of Hybridization Capture Genome Enrichment Methods of ISKNV

Direct sequencing of tissue homogenates and hybridization capture enrichment resulted in identical whole genome sequences for the two samples, which were tested using both methods (Table 2). Genome enrichment of the clinical samples increased read coverage more than 10 times compared to direct sequencing. Enrichment was necessary to obtain a complete genome from samples with less than 1.3 × 10^8^ ISKNV DNA copies mg^−1^ (data not shown). Mapping the probe library to *de novo* assembled genomes showed that sequence data were generated with up to 10% mismatch between the target and probe and despite regions of zero probe coverage (Supplementary Table 1).

### 3.3. ISKNV Genome Assembly and Annotation

The complete ISKNV genome was determined for all 16 samples after target enrichment using the custom SureSelect bait library. This included a range of ISKNV DNA concentration in visceral tissues between 2.5 × 10^5^ and 4.2 × 10^9^ copies.mg^−1^(Table 1). The genomes ranged from 110,394 to 111,666 nucleotides in length with 118–122 predicted ORFs (Table 3). The average depth of coverage for all samples was 1144–4023 reads/nucleotide across all positions. The genomes were most closely related to the ISKNV genotype except for a single sample belonging to the RSIV genotype. The genomes were complete when compared to the representative genomes for the relevant genotype.

### 3.4. Genome Structure and Sequence Identity

Pairwise comparison identified >98.80% nucleotide similarity between 14/16 of the ISKNV genotypes in the study (Table 4) and had >99.8% similarity to previously described genomes from ISKNV Clade 1 (Table 3). One genome (Sample 3) had 96.83–97.36% similarity to the other ISKNV-genotype samples in this study and had 99.95% to a previously described ISKNV-genotype Clade 2 genome (Table 3). There was one genotype (Sample 16) with 92.37–92.55% similarity to other genomes in the study (Table 4) and 93.35% to the ISKNV Clade 1 genome, AFIV-16 (MK689685), but had high similarity (99.75%) to the RSIV Clade 2 genome (KagYT-96, MK689686.1) and therefore was classified as RSIV. A consistent genome structure was observed for all samples with a single super interval identified by MAUVE compared to a representative genome, AFIV-16 (MK689685), and dot plot analyses demonstrated a high degree of collinearity between samples and reference genomes (Supplementary Figure 2).

Annotation identified the consistent presence of predicted core genes from representative ISKNV genomes with >95% amino acid sequence identity (Table 5). Using AFIV (MK689685.1) as a reference genome for annotation, each of the ISKNV genotype samples had 122 ORFs except for 2 samples, with 120 and 121. This was explained by nonsense mutations in ORF 26 and 57 (Sample 3) and ORF 26 (Sample 7). The 14 genomes classified as ISKNV genotype Clade 1 had 92–105 of 122 predicted genes which were 100% identical at the level of amino acid sequence (Table 5). The most variable sequence occurred in ORFs 24, 26, 57, and 58 where pairwise nucleotide identity was below 80%. These ORFs were distinguished by variation in the frequency and sequence of repeated sequences (Supplementary Table 2). There were 120 predicted ORFs for the RSIV genome (Sample 16) which grouped with RSIV Clade 2 and shared 91 ORFs with 100% amino acid identity to RSIV strain KagYT-96 (MK689686.1) (Table 5). This included ORF 109 from RSIV strain KagYT-96, which was absent in PIV 2014a (MK098186). Additionally, ORFs 27 and 52 predicted in PIV 2014a were not included because the annotation criteria were not met (i.e., genes with less than 120 nucleotides). Gene by gene analysis identified 59 core genes (>95% amino acid similarity) for the ISKNV species (Supplementary Table 3) with additional ORFs shared across 2 genotypes: ISKNV and RSIV (8); ISKNV and TRBIV (1) and RSIV and TRBIV (6).

There were 20 and 32 conserved direct repeated sequences in the ISKNV and RSIV genomes, respectively, with 3 shared repeated sequences. The repeated sequences ranged in size from 9 to 222 nucleotides with the frequency between 1.9 and 30.4 (Supplementary Table 2). The repeated sequences differed between clades within the ISKNV and RSIV genotypes with 10/20 of the ISKNV repeats present in Clades 1 and 2 and 7/32 from RSIV present in both Clades of this genotype.

### 3.5. Phylogenetic Analysis

**Figure 1:**
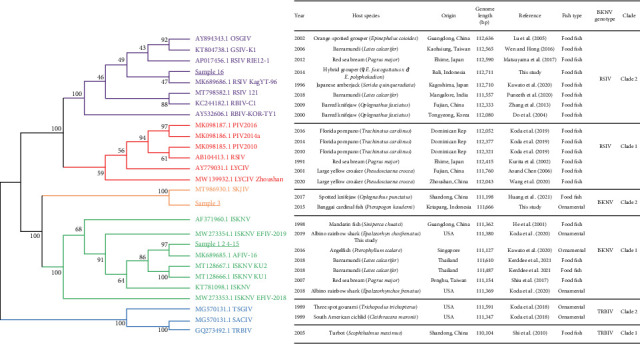
Maximum likelihood phylogenetic cladogram based on 59 ISKNV species core genes and information table for the sequences. Samples 1, 2, 4–15 have identic amino acid sequence for the core genes. The cladogram included 17213 amino-acid sites from 27 whole genome sequences and constructed using Jones-Taylor-Thornton model with empirical frequencies and invariable sites. The analysis was performed using IQ-tree [60] with 1000 bootstraps which applied ultrafast bootstrap [62] after the best model finding using ModelFinder [61]. A bootstrap value of 80 was used as a cutoff point. Alignment using MAFFT with fftns option [66] was performed prior to phylogeny tree construction.

**Figure 2:**
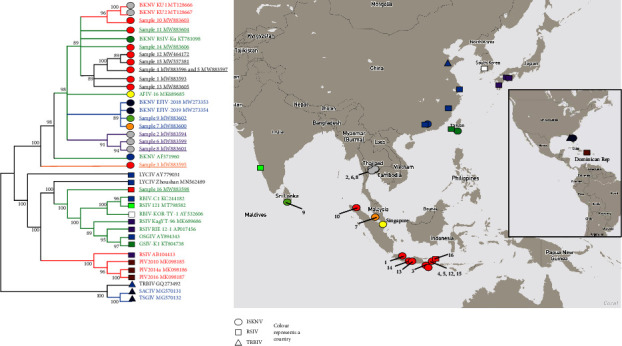
Cladogram of the whole genome sequence of the 16 study samples and ISKNV species based on single nucleotide polymorphism (SNP). The maximum likelihood cladogram was constructed from 39 whole genome sequences including 4701 nucleotide sites on IQ-tree using three substitution type model and unequal base frequencies with the application of ascertainment bias correction and discrete gamma distribution with four rate categories. Bootstraps value of 1000 was applied using ultrafast bootstrap [62] after the best model finding using ModelFinder [61]. Bootstrap value of 80 was used as a cutoff point. SNP alignment was generated using the CSI phylogeny 1.4 web tool [63]. The geographic assignment was performed using Mapbox ©. Circle, square, and triangle represent ISKNV, RSIV, and TRBIV genotypes, respectively. The colours of the symbols represent the country of origin, and the coloured isolate name represents the genotype classification. The numbers on the map indicate the sample ID Number (Table 1).

The phylogenetic analysis using 59 ISKNV-species core genes of published genomes and those from the present study divided the ISKNV species into the three expected genotypes (ISKNV, RSIV, and TRBIV) and supported the two previously identified clades for each genotype (Figure 1). Fourteen of the sixteen study samples were identified within Clade 1 of the ISKNV genotype, and another grouped with Clade 2 of the ISKNV genotype (Figure 1). One sample from grouper aquaculture in Indonesia was identified within Clade 2 of the RSIV genotype (Figures 1 and 2). Interestingly, samples within ISKNV-genotype Clade 1 (Samples 4 and 5) were obtained from hybrids of the same grouper species in the same Indonesian aquaculture location and year as the RSIV genotype (Sample 16).

The use of SNPs for phylogenetic analysis differentiated ISKNV genomes with high similarity and identified patterns of association between viral genome sequence and country of origin, host species and year of the collection (Figure 2). For example, Clade 1 ISKNV genotypes from Indonesia (Samples 1, 4, 5, and 12–15) were grouped separately from samples of the same clade and genotype in Thailand (Samples 2, 6, and 8). Furthermore, there were differences between samples from freshwater aquaculture of giant gourami in Indonesia and examples of the same clade and genotype from marine aquaculture in the same country. Samples from freshwater ornamental fish in Sri Lanka (Sample 9) and Singapore (Sample 7) clustered with ISKNV from the USA, also from freshwater ornamental fish.

## 4. Discussion

This study advanced the knowledge of the ISKNV species by analyzing 16 complete genome sequences and highlighted the importance of considering different genotypes in similar disease scenarios. The hybridization capture enrichment method provided access to viral genomes directly from fish tissues, including those with subclinical infections where the relatively low amount of viral DNA was not previously conducive to whole genome analysis. The sequence similarity within the previously described ISKNV genotype and clade designations was extremely high (>98.8% for ISKNV Clade 1) irrespective of a broad range of hosts, countries of origin, year of collection and clinical statuses of the infected fish. Considerable differences in genomic sequence also added further support for the differentiation of subgroups within the broad ISKNV-species designation to support functional disease control. For example, the increasing availability of sequence data supports the three distinct genotypes of ISKNV despite each causing a disease with similar pathology and an increasing overlap in geographical distribution and range of susceptible fish species [23, 38, 64]. An RSIV-genotype isolate in this study was the probable cause of a clinically indistinguishable disease in hybrid grouper in the same year and location where the disease was caused by the ISKNV genotype. These viruses were readily distinguished with 92.44% nucleotide sequence identity, and only 30 genes with 100% predicted amino acid identity. The RSIV and ISKNV genotypes represent pathogenic virus groups which each have a broad host range but with different genetic properties. Therefore, these and TRBIV should all be considered for functional disease control, such as validation of diagnostic tests, vaccine development and testing for freedom from infection.

The growing number of aquatic pathogens recognized in the genus *Megalocytivirus* across diverse locations and hosts requires genome sequences and associated metadata to be reported according to standard nomenclature. In addition to the distinct genotypes within the species ISKNV, genomic diversity has been reported in SDDV, causing disease in barramundi [65]. Additional megalocytiviruses have been described from freshwater European chub, *Squalius cephalus* [67] and euryhaline three-spine stickleback, *Gasterosteus aculeatus* [68]. There was more than twice the number of core genes (59) within the species ISKNV compared to the 26 core genes identified for the family *Iridoviridae* [69]. Genome analysis revealed that the repeated sequences within ISKNV genomes were consistent at the clade level. The function of the repeated sequence in megalocytiviruses is unknown; however, some studies revealed that repeated sequences influenced protein expression by affecting transcription initiation [70] and mRNA stability [71]. The different repeated sequences in each ISKNV genotype may result in differences in genome regulation which may, in turn, affect their pathogenicity.

The present study highlighted that there was a high level of conservation within the existing genotype and clade designations for ISKNV with >95% amino acid sequence similarity for at least 115 of 122 predicted genes (ISKNV-genotype Clade 1). The nucleic acid target enrichment method demonstrated that this conservation held across clinical and subclinical infections, freshwater and marine fish and isolates obtained several years apart. Hybridization probe libraries enabled the detection of viral genomes with 85–90% sequence mismatches with the library and with sequence gaps of up to 500 nucleotides [72]. In this study, the suitability of the method for identifying sequence diversity was highlighted by the detection of ISKNV Clades 1 and 2 and an RSIV genotype. Other examples of nucleic acid target enrichment have demonstrated the value for determining sequence variation in important aquatic pathogens. Hybridization capture probes successfully enriched a sample limited to 5000 copies of *Cyprinid herpesvirus 3* to determine the 295 kb genome and identified a mixed genotype infection [73]. An alternative enrichment method used oligonucleotide primers with long-range PCR to enrich *Ostreid herpesvirus 1* DNA and identify viral sequence variants from clinical samples [74].

Despite the high similarity of genomes within the species ISKNV, the present method for high-resolution, complete genome sequence determination identified a role for genomic epidemiology. An example of the value of this technique to understand disease occurrence and transmission pathways in aquaculture and determine preventative measures is provided by *Salmon pancreas disease virus* (salmon alphavirus, SAV). The shorter and more variable 12 kb RNA genome was more amenable to such analyzes [75]. However, SNP and nucleotide repeat region analyses of ISKNV might inform disease investigations as the samples within clades that shared a common collection location grouped together on phylogenetic analysis. This will be particularly useful in informing the role of ISKNV spread through international trade in ornamental fish, which is a recognized route for the global spread of aquatic disease agents [76]. This pathway is suspected of having contributed to the increasing distribution of ISKNV, including an incursion into Australia [14, 35]. The present study included an ISKNV-infected dwarf gourami purchased from a pet shop in Sydney, Australia and exported from an unknown Southeast Asian location (Sample 11). Phylogenetic evidence suggested that this location was most likely Indonesia.

Disease caused by ISKNV in fish is multifactorial with important influences from the host and environmental factors on disease expression. For example, disease expression in infected fish was influenced by water temperature [77], although a controlled experimental setting indicated that additional factors also influence the disease outcome [48]. Further studies of ISKNV will benefit from genomic tools to determine viral factors that influence the disease. For example, viral genetic markers of adaptation to freshwater or marine hosts have been suggested [78], and the pathogenicity of a particular ISKNV isolate was reported to be limited to a specific host fish species [79]. Subclinical infection has been documented based on PCR positivity without evaluation of the infective potential of the virus [14]. Access to detailed genomic information directly from hosts with subclinical infection will be the key to differentiating the potential role of viral factors in disease expression.

Important differences between genotypes and further subgroups within the ISKNV species could be overlooked when the host range, environment, and pathology overlap. In the present study, hybrid grouper in Indonesia with similar disease presentations were a source of both RSIV and ISKNV genotypes that could be distinguished by the sequence of the MCP and ATPase genes (95.1% and 96.1% nucleotide identity, respectively). Determining the genotype involved in a disease outbreak is important to indicate appropriate disease control. For example, a commercial vaccine against RSIV (Aquavac IridoV, MSD Animal Health) provided only partial protection against an ISKNV-genotype [11], and cross-protection was not identified between TRBIV and RSIV [79]. This is consistent with a report of the highest antigenicity for RSIV being related to the major capsid protein and the products of ORFs 054L, 055L, 101L, 117L, and 125L [80]. Note that 125L was not identified as an ISKNV core gene with >95% aa similarity. Vaccine development is an area of current research and development interest for ISKNV [81].

Failure to properly differentiate causative pathogens during diagnosis or surveillance for ISKNV will impair efforts for disease control based on biosecurity and vaccination. Therefore, disease control requires validated diagnostic tests for high throughput surveillance for ISKNV that can detect all genotypes as well as differentiate them to guide the appropriate vaccine choice for development. Validation of fit-for-purpose tests for high throughput pan-ISKNV screening assays as well as rapid and convenient tests to differentiate each genotype, is a priority. Such tests are not currently described in the Manual of Diagnostic Tests for Aquatic Animals [17]. The importance of identifying and distinguishing all genotypes of ISKNV is highlighted by disease in mandarin fish being caused by both RSIV and ISKNV [82]. Similarly, the economically and sociocultural important species barramundi is susceptible to RSIV [83], ISKNV [23], and TRBIV [39]. The disease caused by TRBIV in barramundi in Taiwan originated from stock moved internationally and spread to 35 farms across two growing regions [38]. The TRBIV genotype is not currently considered with other ISKNV genotypes as an internationally notifiable pathogen [17]. An additional reason to minimize the spread of different isolates is the potential for ISKNV to undergo recombination [84]. In the related genus *Ranavirus*, natural recombination generated a chimeric pathogen with much higher virulence [85, 86]. Systematic nomenclature for ISKNV will enhance communication for effective regional and international disease management. The genotype classification with a numbering system provides the most functional approach to ISKNV nomenclature and overcomes the confusion of a genotype name overlapping with the virus or host species name i.e., genotypes I (RSIV), II (ISKNV), and III (TRBIV) [9]. For example, a disease report using the virus classification: “The causative agent was identified as Megalocytivirus ISKNV genotype II” [11] was more informative compared to a report with an isolate name: “a new member of megalocytivirus, designated as giant gourami iridovirus”, when the sequence data indicated that the same genotype was present [27].

A thesis has previously been submitted by Fusianto [25] to The University of Sydney.

## 5. Conclusion

Detailed genomic characterization of ISKNV is important to inform disease control for this emerging and WOAH-notifiable pathogen in the aquaculture of food fish and ornamental fish. The broad ISKNV species designation includes readily distinguished genotypes which cause a similar disease with nonspecific clinical signs and overlap in a large number of susceptible fish species. Comparison of complete genomes will identify genomic features associated with distinct viral phenotypes, including virulence factors that contribute to variable disease outcomes in different hosts and environments. A clear nomenclature that supports individual consideration of the subspecies designations of ISKNV is needed to manage fish health. The ISKNV genotypes are sufficiently different from requiring individual consideration in the development of vaccines and validation of diagnostic tests. Control of ISKNV remains a high priority in endemic regions where it is important to limit viral amplification within high-density populations of fish in aquaculture. Whole genome sequences of ISKNV enable the evaluation of the transmission pathways and inform the measures needed to limit the spread of this pathogen.

## Figures and Tables

**Table 1:** Description of samples used to evaluate ISKNV genomic diversity in Southeast Asian aquaculture.

Sample ID	*Collection details*		*Host*		Fish type, habitat	Clinical status	*MCV qPCR result*		Reference
	Country of origin	Year	Species	Common name			Ct (average)	MCV genome copies/mg	
1	Indonesia	2011	*Trichogaster leeri*	Pearl gourami	Ornamental, fresh water	Subclinical	20.38	7.81 × 10^7^	[14]
2	Thailand	2011	*Trichogaster trichopterus*	Blue/gold gourami	Ornamental, fresh water	Subclinical	25.06	2.92 × 10^6^	[14]
3	Indonesia	2015	*Pterapogon kauderni*	Banggai cardinalfish	Ornamental, marine	Subclinical	17.9	4.42 × 10^8^	[44]
4	Indonesia	2014	(♀) *Epinephelus fuscoguttatus* x (♂) *E. polyphekadion*	Hybrid grouper cantik	Food fish, marine	Subclinical	28.57	2.50 × 10^4^	[45]
5	Indonesia	2014	(♀) *E. fuscoguttatus* x (♂) *E. polyphekadion*	Hybrid grouper cantik	Food fish, marine	Subclinical	21.84	2.80 × 10^7^	[45]
6	Thailand	2015	*Trichopodus trichopterus*	Three spot gourami	Ornamental, fresh water	Subclinical	14.57	4.58 × 10^9^	[44]
7	Malaysia	2015	*Xiphophorus helleri*	Green swordtail	Ornamental, fresh water	Subclinical	21.16	4.50 × 10^7^	[44]
8	Thailand	2015	*Trichogaster lalius*	Dwarf gourami	Ornamental, fresh water	Subclinical	16.2	1.46 × 10^9^	[44]
9	Sri Lanka	2015	*Xiphophorus maculatus*	Southern platyfish	Ornamental, fresh water	Subclinical	19.22	1.76 × 10^8^	[44]
10	Indonesia	2015	(♀) *E. fuscoguttatus* x (♂) *E. polyphekadion*	Hybrid grouper	Food fish, marine	Subclinical	24.49	2.72 × 10^7^	[15]
11	SE Asia	2010	*Trichogaster lalius* ^*∗*^	Dwarf gourami	Ornamental, fresh water	Clinical	18.52	2.86 × 10^8^	[46]
12	Indonesia	2016	(♀) *E. fuscoguttatus* x (♂) *E. lanceolatus*	Hybrid grouper cantang	Food fish, marine	Clinical	14.71	4.15 × 10^9^	[25]
13	Indonesia	2018	*Osphronemus goramy*	Giant gourami	Food fish, fresh water	Clinical	24.65	3.91 × 10^6^	This study
14	Indonesia	2018	*Osphronemus goramy*	Giant gourami	Food fish, fresh water	Clinical	16.51	1.17 × 10^9^	This study
15	Indonesia	2016	(♀) *E. fuscoguttatus* x (♂) *E. polyphekadion*	Hybrid grouper cantik	Food fish, marine	Clinical	19.33	1.63 × 10^8^	[47]
16	Indonesia	2014	(♀) *E. fuscoguttatus* x (♂) *E. polyphekadion*	Hybrid grouper cantik	Food fish, marine	Subclinical	24.49	4.37 × 10^6^	[45]

^*∗*^the isolate was sequenced from the 4^th^* in vivo* passage in Murray cod (*Maccullochella peelii*) by Fusianto et al. [48].

**Table 2:** Comparison of ISKNV genomes determined using the hybridization bait capture enrichment method compared to direct sequencing of tissue homogenates for two samples with a high concentration of viral DNA.

Parameter	*Enriched*		*Direct sequencing*	
	Sample 15	Sample 12	Sample 15	Sample 12
Total reads after trimming	2,207,566	3,194,072	8,131,798	5,830,384
Percentage of mapped reads	97.67%	98.44%	1.63%	4.04%
Genome length	110,919	110,961	110,919	110,961
GC content (%)	54.75	54.75	54.75	54.75
Depth of coverage—average	2,508	3,773	163	307
Maximum	6,385	7,525	1,822	1,169
Minimum	391	494	13	73
SNPs (compared to direct sequencing)	0	0		

**Table 3:** Characteristics of the ISKNV genomes determined using a hybrid capture enrichment method for fish tissue homogenates.

Sample ID	Country of origin	Year of collection	Host species	No. of predicted genes	Length (bp)	Average coverage depth	GC content (%)	GenBank Acc	*Closest published MCV genome*		
									Genotype	Percent identity^a^	Acc number
1	Indonesia	2011	Pearl gourami	122	111,309	2682	54.76	MW883593	ISKNV	99.94	AF371960.1
2	Thailand	2011	Blue/gold gourami	122	110,394	2675	54.76	MW883594	ISKNV	99.97	AF371960.1
3	Indonesia	2015	Banggai cardinalfish	120	111,666	3074	54.87	MW883595	ISKNV	99.95	MN432490.1
4	Indonesia	2014	Hybrid grouper, cantik	122	111,039	1918	54.75	MW883596	ISKNV	99.84	MK689685.1
5	Indonesia	2014	Hybrid grouper, cantik	122	111,039	2781	54.75	MW883597	ISKNV	99.84	MK689685.1
6	Thailand	2015	Three spot gourami	122	111,351	4023	54.78	MW883599	ISKNV	99.94	AF371960.1
7	Malaysia	2015	Green swordtail	121	110,927	2661	54.68	MW883600	ISKNV	99.94	AF371960.1
8	Thailand	2015	Dwarf gourami	122	111,014	3266	54.75	MW883601	ISKNV	99.88	MK689685.1
9	Sri Lanka	2015	Southern platyfish	122	111,062	2535	54.73	MW883602	ISKNV	99.84	MK689685.1
10	Indonesia	2015	Hybrid grouper	122	111,138	2430	54.75	MW883603	ISKNV	99.89	MK689685.1
11	SE Asia	2010	Dwarf gourami	122	111,092	3301	54.75	MW883604	ISKNV	99.89	AF371960.1
12	Indonesia	2016	Hybrid grouper, cantang	122	110,961	3773	54.75	MW464172	ISKNV	99.87	AF371960.1
13	Indonesia	2018	Giant gourami	122	111,105	2063	54.77	MW883605	ISKNV	99.94	AF371960.1
14	Indonesia	2018	Giant gourami	122	111,609	3313	54.77	MW883606	ISKNV	99.99	AF371960.1
15	Indonesia	2016	Hybrid grouper, cantik	122	110,919	2509	54.75	MW557381	ISKNV	99.85	MK689685.1
16	Indonesia	2014	Hybrid grouper, cantik	118	112,749	1144	53.14	MW883598	RSIV	99.75	MK689686.1

^a^percent identity derived from the whole genome sequence at the nucleotide level.

**Table 4:** Pairwise comparison of whole genomes characterized in the present study. The nucleotide sequence identity (%) (upper) and genetic distance (lower) were calculated using the maximum composite likelihood model that encompassed 115,409 nucleotides including gaps.

Sample ID	*Sample ID*															
	1	2	3	4	5	6	7	8	9	10	11	12	13	14	15	16
1		99.89	98.26	99.97	99.97	99.96	99.95	99.96	99.96	99.96	99.97	99.97	99.94	99.97	99.98	92.44
2	1.08 × 10^−3^		98.18	99.89	99.89	99.89	99.87	99.88	99.88	99.88	99.88	99.88	99.86	99.89	99.89	92.37
3	1.76 × 10^−2^	1.84 × 10^−2^		98.26	98.26	98.25	98.25	98.25	98.26	98.25	98.26	98.26	98.23	98.26	98.26	92.55
4	2.68 × 10^−4^	1.15 × 10^−3^	1.76 × 10^−2^		100.00	99.95	99.95	99.95	99.95	99.96	99.96	99.99	99.94	99.97	99.99	92.44
5	2.68 × 10^−4^	1.15 × 10^−3^	1.76 × 10^−2^	0		99.95	99.95	99.95	99.95	99.96	99.96	99.99	99.94	99.97	99.99	92.44
6	3.98 × 10^−4^	1.09 × 10^−3^	1.77 × 10^−2^	4.62 × 10^−4^	4.62 × 10^−4^		99.94	99.97	99.95	99.95	99.95	99.95	99.92	99.96	99.96	92.42
7	4.90 × 10^−4^	1.31 × 10^−3^	1.77 × 10^−2^	5.36 × 10^−4^	5.36 × 10^−4^	6.29 × 10^−4^		99.94	99.95	99.94	99.94	99.95	99.92	99.95	99.95	92.42
8	3.98 × 10^−4^	1.18 × 10^−3^	1.77 × 10^−2^	4.62 × 10^−4^	4.62 × 10^−4^	2.96 × 10^−4^	6.29 × 10^−4^		99.95	99.95	99.95	99.95	99.92	99.96	99.96	92.42
9	3.88 × 10^−4^	1.21 × 10^−3^	1.76 × 10^−2^	4.53 × 10^−4^	4.53 × 10^−4^	5.27 × 10^−4^	4.53 × 10^−4^	5.27 × 10^−4^		99.95	99.95	99.95	99.93	99.96	99.96	92.43
10	3.88 × 10^−4^	1.23 × 10^−3^	1.77 × 10^−2^	3.79 × 10^−4^	3.79 × 10^−4^	5.46 × 10^−4^	6.20 × 10^−4^	5.46 × 10^−4^	5.36 × 10^−4^		99.95	99.96	99.93	99.97	99.96	92.43
11	3.14 × 10^−4^	1.16 × 10^−3^	1.76 × 10^−2^	3.79 × 10^−4^	3.79 × 10^−4^	4.72 × 10^−4^	5.64 × 10^−4^	4.72 × 10^−4^	4.62 × 10^−4^	4.62 × 10^−4^		99.96	99.93	99.97	99.96	92.44
12	2.77 × 10^−4^	1.16 × 10^−3^	1.76 × 10^−2^	6.47 × 10^−5^	6.47 × 10^−5^	4.72 × 10^−4^	5.46 × 10^−4^	4.72 × 10^−4^	4.62 × 10^−4^	3.88 × 10^−4^	3.88 × 10^−4^		99.94	99.97	99.99	92.44
13	5.64 × 10^−4^	1.44 × 10^−3^	1.79 × 10^−2^	5.55 × 10^−4^	5.55 × 10^−4^	7.58 × 10^−4^	8.14 × 10^−4^	7.58 × 10^−4^	7.31 × 10^−4^	6.75 × 10^−4^	6.75 × 10^−4^	5.64 × 10^−4^		99.95	99.95	92.41
14	2.68 × 10^−4^	1.09 × 10^−3^	1.76 × 10^−2^	2.59 × 10^−4^	2.59 × 10^−4^	4.25 × 10^−4^	4.81 × 10^−4^	4.25 × 10^−4^	3.98 × 10^−4^	3.42 × 10^−4^	3.42 × 10^−4^	2.68 × 10^−4^	5.36 × 10^−4^		99.98	92.44
15	2.50 × 10^−4^	1.13 × 10^−3^	1.76 × 10^−2^	3.70 × 10^−5^	3.70 × 10^−5^	4.44 × 10^−4^	5.18 × 10^−4^	4.44 × 10^−4^	4.35 × 10^−4^	3.61 × 10^−4^	3.61 × 10^−4^	2.77 × 10^−5^	5.36 × 10^−4^	2.40 × 10^−4^		92.44
16	7.96 × 10^−2^	8.05 × 10^−2^	7.84 × 10^−2^	7.97 × 10^−2^	7.97 × 10^−2^	7.99 × 10^−2^	7.99 × 10^−2^	7.99 × 10^−2^	7.98 × 10^−2^	7.98 × 10^−2^	7.97 × 10^−2^	7.97 × 10^−2^	8.00 × 10^−2^	7.97 × 10^−2^	7.97 × 10^−2^	

**Table 5:** Amino acid similarity for gene-by-gene comparison within the predicted ORFs in the samples from the present study and reference samples from similar genotypes. Data are the sum of genes within the categories for percentage similarity compared to angelfish *iridovirus* AFIV-16 (ISKNV genotype, MK689685), pompano *iridovirus* isolate PIV 2014a (RSIV genotype clade 1, MK098186 PIV 2014), and RSIV isolate KagYT96 (RSIV genotype clade 2, MK689686).

Gene similarity (pairwise amino acid (%))	1	2	3	4	5	6	7	8	9	10	11	12	13	14	15	16
*Compared with ISKNV (MK689685 angelfish iridovirus AFIV-16)*																
100	103	95	30	105	105	93	98	94	101	104	104	105	92	104	105	2
<100–95	14	20	74	12	12	24	19	22	15	12	12	11	25	13	11	56
<95–90	2	3	11	2	2	2	2	2	2	2	2	4	3	2	3	32
<90–85	0	0	0	0	0	1	0	1	1	0	1	0	0	0	0	10
<85–80	0	0	1	0	0	0	0	1	0	0	0	0	0	0	0	2
<80–75	1	0	0	1	1	0	0	0	1	0	0	0	1	0	1	5
<75–70	1	0	1	1	1	0	0	1	1	0	1	0	0	1	1	3
<70	0	3	4	0	0	1	2	0	0	3	1	1	0	1	0	10

*Compared with RSIV clade 1 (MK098186 PIV 2014)*																
100	2	2	1	2	2	2	2	2	2	2	2	2	2	2	2	21
<100–95	56	55	57	56	56	56	56	56	56	56	56	56	54	55	56	82
<95–90	35	34	33	35	35	35	35	34	35	35	35	35	37	36	35	6
<90–85	7	8	9	7	7	7	7	8	7	7	7	8	7	7	8	2
<85–80	5	5	6	5	5	5	5	5	5	4	5	4	5	5	4	2
<80–75	6	6	5	6	6	6	6	6	6	6	6	6	6	6	6	3
<75–70	1	1	1	1	1	2	1	1	1	2	1	1	1	1	1	0
<70	9	10	9	9	9	8	9	9	9	9	9	9	9	9	9	4

*Compared with RSIV clade 2 (MK689686 RSIV isolate KagYT 96)*																
100	2	2	1	2	2	2	2	2	2	2	2	2	2	2	2	107
<100–95	59	59	66	59	59	58	59	59	59	59	59	59	59	59	59	10
<95–90	30	29	25	30	30	30	30	29	30	29	30	30	30	30	30	1
<90–85	14	14	13	14	14	14	14	14	14	14	14	14	13	14	14	0
<85–80	1	1	1	1	1	1	1	1	1	1	1	1	2	1	1	0
<80–75	4	4	5	4	4	5	4	5	4	3	4	4	4	4	4	0
<75–70	1	1	0	1	1	2	1	1	1	2	1	1	1	1	1	0
<70	7	8	7	7	7	6	7	7	7	8	7	7	7	7	7	0

## Data Availability

The data used to support the findings of this study are included within the article and supplementary information files.

## Supplementary Materials

Supplementary Materials Supplementary Table 1. *In silico* evaluation of probe design by mapping to ISKNV reference genomes and comparison with the genomes derived from the hybrid probe enrichment study. No zero-coverage regions were observed after mapping reads produced by Illumina sequencing to the genome produced by de novo assembly. A similarity and length fraction of 0.9 was applied, and global alignment was performed to map the probes and reads using CLC Genomic workbench 12. Supplementary Table 2. Conserved repeated sequence regions identified in ISKNV and RSIV clade 1 and 2. ISKNV genomes mentioned in Figure 1, excluding TRBIV, were analyzed using tandem repeats finder (Benson, 1999). The location of repeated sequences was based on the annotation of Angelfish iridovirus (MK689685). Supplementary Table 3. Core genes of members of the species ISKNV after a cutoff point of 95% amino acid identity across ISKNV genotypes. Supplementary Figure 1. Hybridization probe design for ISKNV genome enrichment from fish tissue samples. The design used a nucleotide alignment of 12 ISKNV genomes representing the 2 Clades within each of three genotypes to identify conserved regions (A) where nucleotide similarity was >90% in regions of 200 bases or variable regions (B) with <90% similarity within 200 base regions. The 120 bp hybridization capture probes were tiled for 2x coverage across the conserved regions, and unique probes were designed for each unique sequence within the variable regions to ensure >2 coverage. Supplementary Figure 2. Dot plots for pairwise comparison between samples: (A) Sample 1 (representing the ISKNV Clade 1 samples in this study) with MK689685 Angelfish iridovirus AFIV-16; (B) Sample 16 (RSIV genotype clade 2) and MK098186.1 Pompano iridovirus isolate PIV 2014a; (C) Sample 5 (ISKNV genotype Clade 1) and Sample 16 (RSIV genotype) which were obtained from the same aquaculture site and time but were amongst the most dissimilar genomes detected in this study.
