# Supplementary material for: Genotypic Characterization of Infectious Spleen and Kidney Necrosis Virus (ISKNV) in Southeast Asian Aquaculture
Source: Transbound Emerg Dis. 2023 Mar 21;2023:6643006. doi: 10.1155/2023/6643006 (PMC12017167; doi:10.1155/2023/6643006)
Supplement: Supplementary Materials — Supplementary Table 1. In silico evaluation of probe design by mapping to ISKNV reference genomes and comparison with the genomes derived from the hybrid probe enrichment study. No zero-coverage regions were observed after mapping reads produced by Illumina sequencing to the genome produced by de novo assembly. A similarity and length fraction of 0.9 was applied, and global alignment was performed to map the probes and reads using CLC Genomic workbench 12. Supplementary Table 2. Conserved repeated sequence regions identified in ISKNV and RSIV clade 1 and 2. ISKNV genomes mentioned in Figure 1, excluding TRBIV, were analyzed using tandem repeats finder (Benson, 1999). The location of repeated sequences was based on the annotation of Angelfish iridovirus (MK689685). Supplementary Table 3. Core genes of members of the species ISKNV after a cutoff point of 95% amino acid identity across ISKNV genotypes. Supplementary Figure 1. Hybridization probe design for ISKNV genome enrichment from fish tissue samples. The design used a nucleotide alignment of 12 ISKNV genomes representing the 2 Clades within each of three genotypes to identify conserved regions (A) where nucleotide similarity was >90% in regions of 200 bases or variable regions (B) with <90% similarity within 200 base regions. The 120 bp hybridization capture probes were tiled for 2x coverage across the conserved regions, and unique probes were designed for each unique sequence within the variable regions to ensure >2 coverage. Supplementary Figure 2. Dot plots for pairwise comparison between samples: (A) Sample 1 (representing the ISKNV Clade 1 samples in this study) with MK689685 Angelfish iridovirus AFIV-16; (B) Sample 16 (RSIV genotype clade 2) and MK098186.1 Pompano iridovirus isolate PIV 2014a; (C) Sample 5 (ISKNV genotype Clade 1) and Sample 16 (RSIV genotype) which were obtained from the same aquaculture site and time but were amongst the most dissimilar genomes detected in this study. [file 6643006.f1.docx]

**Supplementary Table 1.** *In silico* evaluation of probe design by mapping to ISKNV reference genomes and comparison with the genomes derived from the hybrid probe enrichment study. No zero-coverage regions were observed after mapping reads produced by Illumina sequencing to the genome produced by de novo assembly. A similarity and length fraction of 0.9 were applied and global alignment was performed to map the probes and reads using CLC Genomic workbench 12.

| Sample/ Reference ID | Mapped probes | Unmapped probes |  | Coverage (exclude zero coverage) | | | |  | Zero coverage region | | | |  | Illumina sequencing results | | | |  |
| --- | --- | --- | --- | --- | --- | --- | --- | --- | --- | --- | --- | --- | --- | --- | --- | --- | --- | --- |
|  |  |  |  | Max | Average | Min | St dev |  | Total Number | min | max | Total size (bp) |  | Max | Average | Min | St dev | |
| 1 | 2,062 | 659 |  | 7 | 2.22 | 1 | 0.95 |  | 5 | 1 | 70 | 100 |  | 4,349 | 2,682 | 362 | 554 | |
| 2 | 2,035 | 686 |  | 6 | 2.22 | 1 | 0.94 |  | 6 | 1 | 92 | 192 |  | 4,807 | 2,675 | 329 | 637 | |
| 3 | 2,050 | 671 |  | 7 | 2.24 | 1 | 0.97 |  | 25 | 1 | 158 | 1,613 |  | 5,338 | 3,074 | 286 | 866 | |
| 4 | 2,053 | 668 |  | 6 | 2.22 | 1 | 0.95 |  | 7 | 1 | 95 | 206 |  | 3,321 | 1,918 | 250 | 449 | |
| 5 | 2,053 | 668 |  | 6 | 2.22 | 1 | 0.95 |  | 7 | 1 | 95 | 206 |  | 4,453 | 2,781 | 331 | 590 | |
| 6 | 2,051 | 670 |  | 6 | 2.21 | 1 | 0.94 |  | 7 | 1 | 104 | 227 |  | 7,656 | 4,023 | 30 | 950 | |
| 7 | 2,044 | 677 |  | 6 | 2.21 | 1 | 0.95 |  | 6 | 1 | 205 | 305 |  | 5,747 | 2,661 | 235 | 773 | |
| 8 | 2,050 | 671 |  | 6 | 2.22 | 1 | 0.94 |  | 6 | 1 | 70 | 126 |  | 6,531 | 3,266 | 469 | 785 | |
| 9 | 2,046 | 675 |  | 6 | 2.22 | 1 | 0.94 |  | 7 | 1 | 70 | 122 |  | 4,217 | 2,535 | 241 | 544 | |
| 10 | 2,048 | 673 |  | 6 | 2.21 | 1 | 0.94 |  | 6 | 1 | 86 | 186 |  | 5,445 | 2,430 | 365 | 750 | |
| 11 | 2,048 | 673 |  | 6 | 2.22 | 1 | 0.94 |  | 7 | 1 | 165 | 266 |  | 6,444 | 3,301 | 513 | 751 | |
| 12 | 2,054 | 667 |  | 6 | 2.23 | 1 | 0.95 |  | 6 | 1 | 95 | 205 |  | 7,525 | 3,773 | 494 | 1,109 | |
| 13 | 2,054 | 667 |  | 8 | 2.22 | 1 | 0.95 |  | 6 | 1 | 70 | 164 |  | 4,986 | 2,063 | 172 | 585 | |
| 14 | 2,052 | 669 |  | 6 | 2.21 | 1 | 0.95 |  | 8 | 1 | 481 | 661 |  | 6,318 | 3,313 | 20 | 905 | |
| 15 | 2,050 | 671 |  | 6 | 2.22 | 1 | 0.94 |  | 7 | 1 | 95 | 206 |  | 6,385 | 2,509 | 391 | 671 | |
| 16 | 2,150 | 597 |  | 6 | 2.22 | 1 | 0.94 |  | 10 | 1 | 98 | 168 |  | 7,280 | 2,065 | 34 | 626 | |
| AF371960 ISKNV | 2,059 | 662 |  | 7 | 2.22 | 1 | 0.95 |  | 6 | 1 | 70 | 104 |  |  |  |  |  | |
| KT781098 ISKNV RSIV-Ku | 2,062 | 659 |  | 7 | 2.23 | 1 | 0.94 |  | 5 | 1 | 18 | 34 |  |  |  |  |  | |
| AY532606 RBIV-KOR-TY1 | 2,120 | 601 |  | 6 | 2.28 | 1 | 0.93 |  | 26 | 1 | 111 | 353 |  |  |  |  |  | |
| AB104413 RSIV | 2,103 | 618 |  | 6 | 2.25 | 1 | 0.94 |  | 15 | 1 | 73 | 442 |  |  |  |  |  | |
| AP017456 RSIV-RIE 12-1 | 2,143 | 578 |  | 6 | 2.29 | 1 | 0.93 |  | 11 | 1 | 98 | 253 |  |  |  |  |  | |
| KT804738 GSIV-K1 | 2,143 | 578 |  | 6 | 2.29 | 1 | 0.93 |  | 10 | 1 | 98 | 188 |  |  |  |  |  | |
| AY779031 LYCIV | 2,068 | 653 |  | 6 | 2.24 | 1 | 0.93 |  | 23 | 1 | 127 | 1,023 |  |  |  |  |  | |
| AY894343 OSGIV | 2,143 | 578 |  | 6 | 2.28 | 1 | 0.93 |  | 10 | 1 | 98 | 253 |  |  |  |  |  | |
| KC244182 RBIV C1 | 2,139 | 582 |  | 6 | 2.29 | 1 | 0.93 |  | 9 | 1 | 98 | 187 |  |  |  |  |  | |
| GQ273492 TRBIV | 1,842 | 879 |  | 6 | 2.05 | 1 | 0.91 |  | 42 | 1 | 203 | 2,296 |  |  |  |  |  | |
| MG570132 TSGIV | 1,762 | 959 |  | 6 | 1.98 | 1 | 0.88 |  | 76 | 1 | 299 | 4,761 |  |  |  |  |  | |
| MG570131 SACIV | 1,765 | 956 |  | 6 | 1.97 | 1 | 0.89 |  | 70 | 1 | 184 | 4,067 |  |  |  |  |  | |

**Supplementary Table 2.** Conserved repeated sequence regions identified in ISKNV and RSIV clade 1 and 2. ISKNV genomes mentioned in Figure 1 excluding TRBIV were analyzed using tandem repeats finder (Benson, 1999). The location of repeated sequences was based on annotation of Angelfish iridovirus (MK689685).

| Consensus pattern | Length (bp) | Location | ISKNV | | | | |  | RSIV | | | | |
| --- | --- | --- | --- | --- | --- | --- | --- | --- | --- | --- | --- | --- | --- |
|  |  |  | Clade 1 | |  | Clade 2 | |  | Clade 1 | |  | Clade 2 | |
|  |  |  | Match identity (%) | Frequency |  | Match identity (%) | Frequency |  | Match identity (%) | Frequency |  | Match identity (%) | Frequency |
| GACCGGCGCTTGGCGGGC | 18 | Orf 20 | 86-95 | 4.6-5.7 |  | 97 | 3.6 |  | 86-90 | 4.9-5.9 |  | 86 | 4.6 |
| GACCGGCGCTTGGCGGGCGACCTGCGCTTGGCGGGC | 36 | Orf 20 | 87 | 2.7 |  | - | - |  | - | - |  | 85 | 2.3 |
| CGGGCGACCTGCGCTTGG | 18 | Orf 20 | 92 | 4.7 |  | - | - |  | - | - |  | 89 | 2.6 |
| TGGTCAATATGATGTTACTACCACTGAACCATGAGTGCTAAT… | 47 | Orf 24 | 100 | 2 |  | 100 | 2 |  | - | - |  | - | - |
| GGCGCTGGT | 9 | Orf 23 | - | - |  | 88 | 8 |  | - | - |  | - | - |
| GGCGCTGGTGGCGCTGGTGGCGCCGGT | 27 | Orf 23 | - | - |  | 93 | 2.7 |  | - | - |  | - | - |
| ACAGGCCTGACAGGCCTGACGCGCACC | 27 | Orf 26 | 65-85 | 8.9-30.4 |  | 65-66 | 15-37 |  | - | - |  | - | - |
| GACGCGCACCACAGGCCTGACGCGCACCACAGGCCTGACAC… | 45 | Orf 26 | - | - |  | 77-89 | 5.5-18.5 |  | - | - |  | - | - |
| ACAGGCCTG | 9 | Orf 26 | - | - |  | 100 | 3.3 |  | - | - |  | - | - |
| ACGACGCGCACCACAGGCCTGACAGGCCTGACACGC | 36 | Orf 26 | 72-100 | 3.6-18.7 |  | 87 | 4.7 |  | - | - |  | - | - |
| ACAGGCCTGACACGCACC | 18 | Orf 26 | 95-100 | 1.9-3.4 |  | 94 | 2 |  | - | - |  | - | - |
| AAGACGGCAGTTTATTGAGTTGTTACACATATAATATTAGCCA… | 88 | Orf 32 | - | - |  | 98 | 2.3 |  | - | - |  | - | - |
| AGGGTCGCCACGTCGCAGAGGAACACGAGCCTCCCTTG… | 75 | Orf 57 | 97 | 1.9 |  | - | - |  | - | - |  | - | - |
| GCTGCGTAGTGGATGTCATGGACCATCGTGGCTACG | 36 | Orf 58 | 97 | 2 |  | 97 | 2 |  | - | - |  | - | - |
| AGTGGATGTCATGGACCATCGTGGCTACGGCTGCAC | 36 | Orf 58 | 88 | 2.1 |  | 88 | 2.1 |  | - | - |  | - | - |
| TTGGTCCATGTCGCGCGGGTCCTCCGGTTCAGGCAGGCGTCTG… | 183 | Orf 60 | - | - |  | 96 | 2 |  | - | - |  | - | - |
| TTAATCATACACAGCACACCACAAAGTATGTACAC | 35 | Orf 64-65 | 94 | 2 |  | 97 | 2 |  | - | - |  | - | - |
| GGTGCCCGAAGAGGTACGCC | 21 | Orf 87 | 86 | 2 |  | 86 | 2 |  | - | - |  | - | - |
| GATGGCAGCGGTGCGTCGGTTCCAACGGTA | 30 | Orf 92 | 96 | 1.9 |  | 96 | 1.9 |  | - | - |  | - | - |
| GACGTGCCCACTCCCGCAAGGACAACATATGTGGTGCCTC… | 96 | Orf 114 | 94 | 2.2 |  | - | - |  | - | - |  | - | - |
| GGCACCGGAGATGGTGGTCGCGCTGGCACCGGAACTGGTACC | 42 | Orf 23 | - | - |  | - | - |  | - | - |  | 97 | 1.9 |
| CCTGAGGAACCGGAGGAAGAAGAGGACGAGTACGACTGTCC… | 174 | Orf 24 | - | - |  | - | - |  | - | - |  | 75 | 3.6 |
| ACCACCACCACTGTGGCACCTACAACA | 27 | Orf 24 | - | - |  | - | - |  | - | - |  | 95 | 2.7 |
| ACAGGCCCGACGCGCACC | 18 | Orf 26 | - | - |  | - | - |  | - | - |  | 82 | 8.4-30.4 |
| GGTGCTGGCACC | 12 | Orf 23 | - | - |  | - | - |  | 84 | 4.2 |  | - | - |
| GCACCGGTGCTGGCA | 15 | Orf 23 | - | - |  | - | - |  | 86 | 3 |  | - | - |
| GCTGGCACCGGAGATGGTGGTCGC | 24 | Orf 23 | - | - |  | - | - |  | 100 | 2.5 |  | - | - |
| GAGCCTGAAGAACCAGAGGAAGAAGAGGACGAGTACGACT… | 171 | Orf 24 | - | - |  | - | - |  | 86 | 1.9 |  | - | - |
| ACCACCACCACTGTGGCACACACA | 27 | Orf 26 | - | - |  | - | - |  | 90 | 3.5 |  | - | - |
| CAACCACCACCACCACTGTGGCACCTACAA | 27 | Orf 26 | - | - |  | - | - |  | 89 | 2.8 |  | - | - |
| ACCACCACCACTGTGGCACCTACAACAACCACCACCACTGTG… | 222 | Orf 24 | - | - |  | - | - |  | 92 | 2 |  | - | - |
| ACCACCACTGTGCCACCTACAACAACACCACCACTGTGGCAC… | 168 | Orf 24 | - | - |  | - | - |  | 93 | 1.9-2 |  | 92-93 | 1.9-2 |
| CACACCACAGGCCTGACAGGCCCGACG | 27 | Orf 26 | - | - |  | - | - |  | 71-76 | 11.5-13.5 |  | - | - |
| ACAGGCCCGACGCACACCACAGGCCTGACAGGCCCGACGCA… | 45 | Orf 26 | - | - |  | - | - |  | 73-96 | 4.2-7.6 |  | - | - |
| ACAGGCCCGACGCACGCCACAGGCCCGACGCACGACGCACG… | 72 | Orf 26 | - | - |  | - | - |  | 85 | 3-3.5 |  | - | - |
| ACAGGCCCGACGCACGCC | 18 | Orf 26 | - | - |  | - | - |  | 68-100 | 4.4-11.4 |  | - | - |
| ACCCCCGCCTGTTAGGCTAAGAAGGGTCGCCACGTTGCAGAG… | 76 | Between orf 56-57 | - | - |  | - | - |  | 99 | 2.6 |  | - | - |
| GACCCCCGCCTGTTAGGCTAAGAAGGGTCGCCACGTTGCAGA… | 55 | Between orf 56-57 | - | - |  | - | - |  | 90 | 3.8-4.8 |  | - | - |
| GGCACATGCGTCGACCCGGCGT | 22 | Orf 54 | - | - |  | - | - |  | 100 | 2.4-3.4 |  | 85 | 1.9 |
| TAGCGGCGACCCCCGCCTGTTAGGCTAAGAAGGGTCGCCACG… | 69 | Between orf 56-57 | - | - |  | - | - |  | - | - |  | 97 | 2.5 |
| GCCACCGGTCATCTGGCCATATTCAAATATTTCCCAATATCAC… | 66 | Between orf 56-57 | - | - |  | - | - |  | 83 | 2.5 |  | - | - |
| CACATATATCAATATACCCAGCCACCGATCATCTGGCCATAT… | 66 | Between orf 56-57 | - | - |  | - | - |  | 88 | 1.9 |  | - | - |
| GCGTCTGAAGGGGCTGTCAGTCTCACTTGGCTCGTCCTGCAGG… | 57 | Orf 60 | - | - |  | - | - |  | - | - |  | 82 | 2 |
| TGCAGGGGCCGCTCAGGCAAGTCATCT | 27 | Orf 60 | - | - |  | - | - |  | 73 | 5.3 |  | 97 | 2.3 |
| GTGCGACACCGTGCCATAGACGACAGTCCCCAGCACATGGG… | 57 | Orf 65 | - | - |  | - | - |  | 82 | 2.2 |  | 82 | 2.2 |
| GGTCAGGCTTTGGTG | 15 | Orf 88 | - | - |  | - | - |  | 88 | 6.9 |  | 88 | 7.9 |
| CACCTGTACAATGCATGTGTGAGAATCAAAATGTGCAACACT… | 94 | Orf 114 | - | - |  | - | - |  | - | - |  | 100 | 2 |
| CAATTTGCATCAAGTGACTTTATTTCTACATTGTATTG | 38 | Between orf 103-104 | - | - |  | - | - |  | - | - |  | 100 | 2 |
| CACCACCTTACCAGCCGCCTGCTATTCCACCCA | 33 | Orf 114 | - | - |  | - | - |  | 92 | 22 |  | 96 | 1.9 |

**Supplementary Table 3**. Core genes of members of the species ISKNV after a cutoff point of 95% amino acid identity across ISKNV genotypes.

| ORF^a^ | Putative function | Position | | Strand +/- |
| --- | --- | --- | --- | --- |
|  |  | Start | Stop |  |
| 1 | Transmembrane amino acid transporter protein | 134 | 1270 | - |
| 2 | DNA dependent RNA polymerase subunit H-like protein | 1240 | 1695 | - |
| 7 | Major capsid protein | 3794 | 5155 | - |
| 8 | Myristylated membrane protein | 5174 | 6631 | - |
| 10 | Unknown | 8342 | 8503 | + |
| 11 | Unknown | 8662 | 9054 | - |
| 12 | Unknown | 9051 | 9311 | - |
| 13 | RING-finger-containing E3 ubiquitin ligase | 9330 | 9662 | + |
| ORF15^b^ | Unknown | 11017 | 11394 | - |
| 15 | Unknown | 11309 | 12268 | + |
| 16 | Unknown | 12278 | 13069 | + |
| 17 | Unknown | 13129 | 13716 | - |
| 20 | DNA polymerase | 14579 | 17425 | + |
| 21 | Unknown | 17467 | 18006 | + |
| 24 | Ribonucleotide diphosphate reductase small subunit | 22300 | 23238 | + |
| 27 | DNA repair protein RAD2 | 23942 | 24838 | - |
| 28 | DNA dependent RNA polymerase largest subunit | 24855 | 28361 | - |
| 29 | Transcription factor S-II | 28368 | 28589 | - |
| 33 | DNA dependent RNA polymerase second largest subunit | 30919 | 34053 | + |
| 42 | Thiol oxidoreductase | 43255 | 43617 | - |
| 45 | Cytosine DNA methyltransferase | 45333 | 46016 | - |
| 46 | Unknown | 46176 | 46439 | + |
| 48 | Unknown | 46796 | 46966 | + |
| 49 | Unknown | 47025 | 47453 | - |
| 54 | Unknown | 50294 | 50941 | - |
| 55 | Unknown | 50948 | 51208 | - |
| 58 | Replication factor | 52134 | 52937 | - |
| 60 | Unknown | 56620 | 56988 | - |
| 61 | SNF2 family helicase | 57002 | 59650 | - |
| 62 | mRNA capping enzyme | 59693 | 61168 | - |
| 66 | Unknown | 63671 | 65104 | - |
| 69 | Unknown | 66207 | 67817 | - |
| 74 | Unknown | 70820 | 73792 | - |
| 75 | Ankyrin repeat containing protein | 73810 | 75144 | + |
| 76 | Unknown | 75141 | 75605 | + |
| 78 | Unknown | 76142 | 76639 | + |
| 79 | Unknown | 76676 | 77782 | - |
| 80 | Unknown | 77834 | 78193 | + |
| 84 | Ribonuclease III | 80715 | 81485 | + |
| 85 | SAP domain-containing protein | 81482 | 81895 | - |
| ORF88^b^ | Unknown | 81939 | 83495 | + |
| 91 | Putative RNA binding protein | 84635 | 85561 | - |
| 92 | Unknown | 85571 | 86071 | - |
| 94 | Unknown | 87264 | 88073 | - |
| 95 | Unknown | 88007 | 88498 | - |
| 98 | Unknown | 89511 | 90026 | - |
| 101 | Unknown | 91990 | 92766 | + |
| 102 | Unknown | 92768 | 93133 | + |
| 105 | D5 family NTPase | 94949 | 97714 | - |
| 106 | Unknown | 97761 | 97916 | + |
| 108 | Proliferating cell nuclear antigen-like protein | 98823 | 99566 | + |
| 109 | Unknown | 99556 | 100059 | - |
| 111 | FV3 immediate-early protein ICP46-like protein | 102968 | 103978 | + |
| 114 | FV3 31KDa-like protein | 105486 | 106160 | - |
| 116 | RING-finger-containing E3 ubiquitin ligase | 107870 | 108157 | + |
| 117 | Unknown | 108189 | 108698 | + |
| 119 | ATPase | 109359 | 110078 | + |
| 120 | Unknown | 110050 | 110421 | - |
| 121 | Ankyrin repeat-containing protein | 110430 | 111116 | - |

^a^ ORF designations were based on the positions within the genome of Angelfish iridovirus strain AFIV-16 (MK689685) except two ORFs

^b^ ORF from Pompano iridovirus PIV2014a (MK098186)


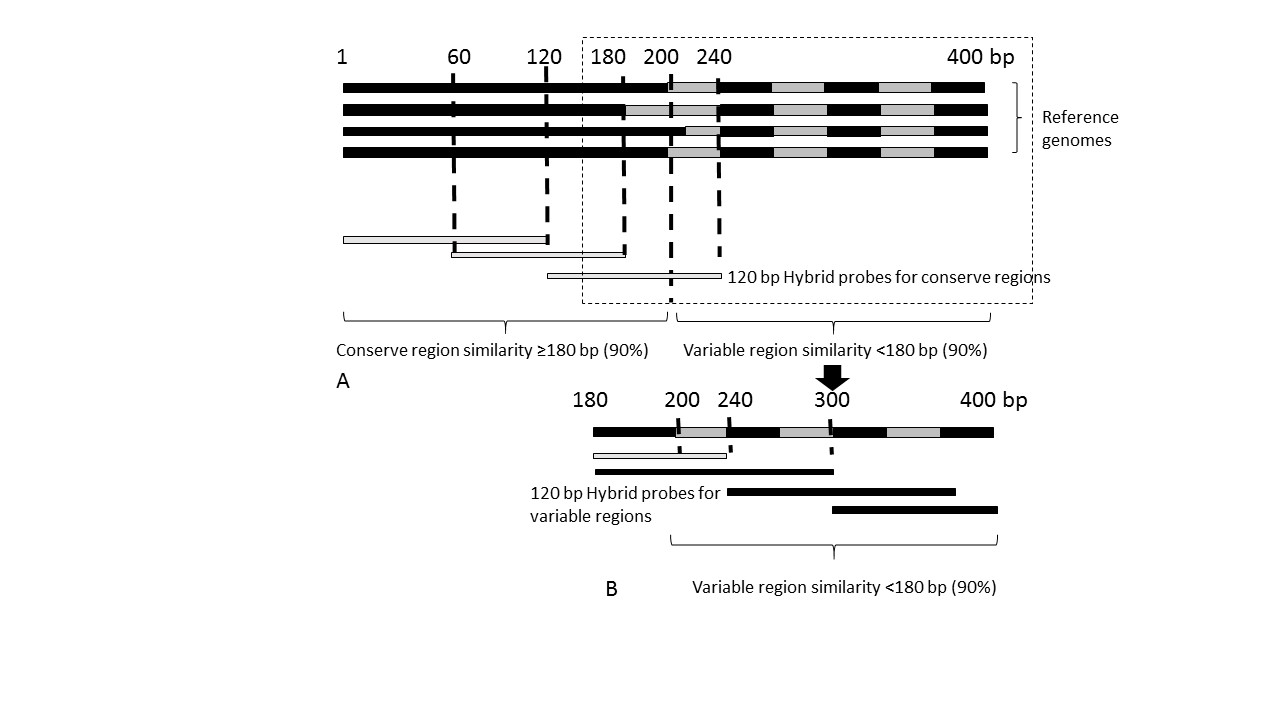


**Supplementary Figure 1**. Hybridization probe design for ISKNV genome enrichment from fish tissue samples. The design used a nucleotide alignment of 12 ISKNV genomes representing the 2 Clades within each of three genotypes to identify conserved regions (A) where nucleotide similarity was >90% in regions of 200 bases, or variable regions (B) with <90% similarity within 200 base regions. The 120 bp hybridization capture probes were tiled for 2x coverage across the conserved regions and unique probes were designed for each unique sequence within the variable regions to ensure >2 coverage.


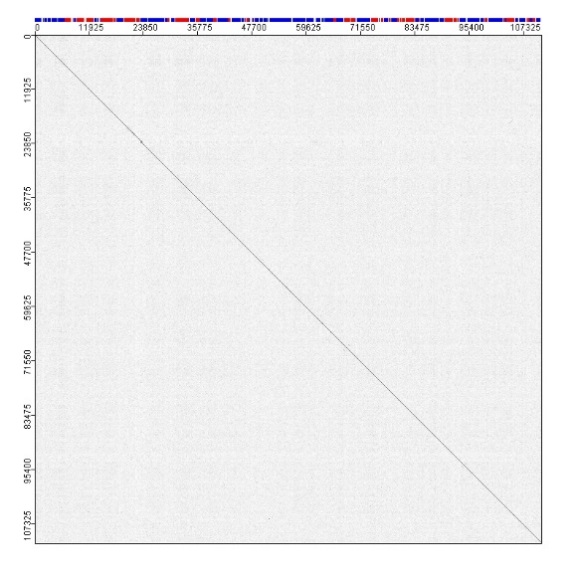

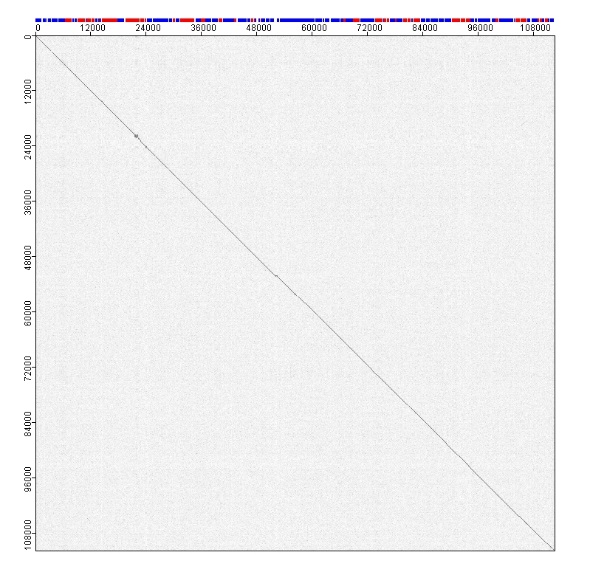

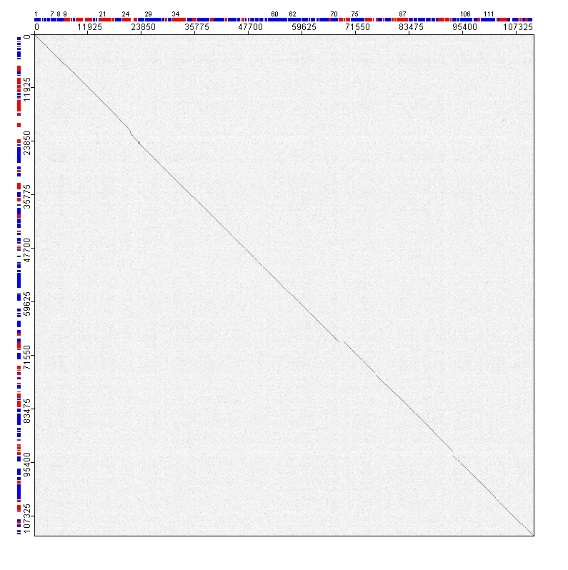


A B

C

**Supplementary Figure 2**. Dot plots for pairwise comparison between samples: A. Sample 1 (representing the ISKNV Clade 1 samples in this study) with MK689685 Angelfish iridovirus AFIV-16; B. Sample 16 (RSIV genotype clade 2) and MK098186.1 Pompano iridovirus isolate PIV 2014a; C. Sample 5 (ISKNV genotype Clade 1) and Sample 16 (RSIV genotype) which were obtained from the same aquaculture site and time but were amongst the most dissimilar genomes detected in this study.
